# Supplementary material for: A scoping review of determinants of performance in dressage
Source: PeerJ. 2020 Apr 24;8:e9022. doi: 10.7717/peerj.9022 (PMC7185025; doi:10.7717/peerj.9022)
Supplement: Table S1 [file peerj-08-9022-s001.docx]

Supplemental Table S1: The Scales of Training (adapted from British Dressage, 2019)

| Measurement | Definition |
| --- | --- |
| Rhythm | Regular, that is correct for each pace and at the same tempo with a pronounced beat. |
| Relaxation | Suppleness and elasticity, free from resistance. |
| Contact/connection | Light, even and elastic contact, working though from the hind limbs. Indicates a level of throughness with the horse functioning in one piece. |
| Impulsion | Elastic loading and unloading of the limbs during stance that springs the horse off the ground. Indicates the amount of energy being created and contained, but without resistance. |
| Straightness | Symmetrical propulsive power in both hind limbs, symmetrical lifting and stabilization by both forelimbs and symmetrical contact (mirrored on left and right turns/circles) in the left and right reins. |
| Collection | Control of the horse’s trunk rotations and maintenance of an uphill trunk orientation. Greater weight distributed to the hind limbs from the forelimbs. |
