# Supplementary material for: A scoping review of determinants of performance in dressage
Source: PeerJ. 2020 Apr 24;8:e9022. doi: 10.7717/peerj.9022 (PMC7185025; doi:10.7717/peerj.9022)
Supplement: Table S2 [file peerj-08-9022-s002.docx]

**Study inclusion criteria for “A scoping review of determinants of performance in dressage”**

| Population | - Horse rider/ Equestrian - Elite and Non-Elite - Able-bodied and Impaired - Horse - Judges (Dressage, Breeding, Showing) |
| --- | --- |
| Setting | - Competition - Experimental (Ridden Horse or Simulator) - Breed Evaluation - Therapeutic Riding |
| Outcome | - Quantitative (Kinematic, Kinetic, EMG, Postural, COP/ Pressure, Strain Gauge, Timing/ Coordination) - Scores (i.e. judging) |
