# Supplementary material for: A scoping review of determinants of performance in dressage
Source: PeerJ. 2020 Apr 24;8:e9022. doi: 10.7717/peerj.9022 (PMC7185025; doi:10.7717/peerj.9022)
Supplement: Table S8 [file peerj-08-9022-s008.docx]

Supplemental Table S7: Significant horse performance outcome measures

| **Outcome Measures**  **(Measurement Tool)** | **Gait/ Movement** | **Articles** | **Significant Performance Effect** |
| --- | --- | --- | --- |
| **TEMPORAL MEASURES** | | | |
| Stride Duration (Kinematics) | Trot, extended trot | Holmstrom et al. (1994), Deuel and Park (1990b) | Longer stride duration |
| FL Stride Duration (Kinematics) | Trot (treadmill) | Back et al. (1994) | Longer FL stride duration |
| FL Stance Duration (Kinematics) | Extended trot, one-stride lead change | Deuel and Park (1990a, b) | Shorter FL stance duration |
| HL Stance Duration (Kinematics) | Trot, passage, piaffe | Holmstrom et al. (1994), Clayton (1997) | Shorter HL stance duration (% stride) |
| Swing Duration (Kinematics) | Extended trot, one-stride lead change, two-stride lead change (pre-change stride) | Deuel and Park (1990a, b) | Shorter HL and longer FL swing duration |
| Stride frequency (Accelerometer) | Walk, trot | Biau and Barrey (2004) | Longer |
| Stride Regularity (Accelerometer) | Walk, trot, canter | Biau and Barrey (2004) | Greater |
| Stride Symmetry (Accelerometer) | Walk | Biau and Barrey (2004) | Greater |
| Suspension/ Airborne duration (Kinematics) | Trot-halt transition, halt-trot transition, one-stride lead change | Tans et al. (2009), Deuel and Park (1990a) | Longer Suspension Duration |
| Hind first diagonal dissociation  (Kinematics) | Trot, collected trot, passage, piaffe, trot-halt transition, halt-trot transition | Clayton (1997), Holmstrom et al. (1994), Tans et al. (2009) | Positive (HL impacts first) |
| Hind first diagonal lift off (Kinematics) | Trot-halt transition, halt-trot transition | Tans et al. (2009) | Positive (HL impacts first) |
| Forelimb Duty Factor (Kinematics) | Trot, trot-halt transition, halt-trot transition | Holmstrom et al. (1994), Tans et al. (2009) | Smaller FL % |
| Hindlimb Duty Factor (Kinematics) | Trot, trot-halt transition, halt-trot transition | Holmstrom et al. (1994), Tans et al. (2009) | Smaller HL % |
| Tripedal Contact Duration (Kinematics) | Extended canter (left lead), one-stride lead change | Deuel and Park, (1990a) | Shorter HL tripedal contact duration |
| Unipedal Contact Duration (Kinematics) | Two-stride lead change (pre-lead change stride) | Deuel and Park, (1990a) | Shorter TrF contact |
| Impact Interval (Kinematics) | Extended trot | Deuel and Park (1990b) | Right lateral limbs and right HL - left FL impact intervals had significant relationship with Team score. For Finalists, score positively related to FL impact interval and Right lateral limb interval and left HL-right FL interval. |
| Transition duration (Kinematics) | Trot-walk transition, canter-halt transition, canter-trot transition | Biau et al. (2002) | Longer duration |
| Presence of intermediate steps (Kinematics) | Walk-trot transition, trot-walk transition | Argue and Clayton (1993a) | Transitions without intermediate steps |
| **JOINT/SEGMENT KINEMATICS** | | | |
| Head Angle - Behind Vertical <90 degrees (Kinematics) | Piaffe | Lashley et al. 2014 | More behind vertical |
| Forelimb Pro/ Retraction (Kinematics) | Trot, in-hand trot | Holmstrom et al. 1994, Morales et al. 1998 | Trot: greater ROM and greater FL swing phase angle in retraction. In-hand: smaller ROM due to significantly smaller retraction angle. |
| Hindlimb Pro/ Retraction (Kinematics) | Trot, in-hand trot | Holmstrom et al. 1994, Morales et al. 1998 | Greater ROM with a greater protraction angle |
| Elbow Joint motion (Kinematics) | Trot, in-hand trot | Holmstrom et al. 1994, Morales et al. 1998 | Trot: increased flexion at beginning of swing phase. In-hand trot: less flexed at lift-off. |
| Carpal Joint motion (Kinematics) | Trot, in-hand trot | Holmstrom et al. 1994, Morales et al. 1998 | Trot: increased flexion at beginning of swing phase. In-hand trot: greater maximum and minimum angles and greater angles at lift-off, landing and peak flight arc. |
| Forelimb Fetlock Joint Motion (Kinematics) | Trot (treadmill), in-hand trot | Back et al. (1994), Morales et al. (1998) | In-hand: greater extension at lift-off, landing and peak flight arc. |
| Tarsal Joint Motion (Kinematics) | Trot | Holmstrom et al. 1994 | Increased flexion at stance |
| Hindlimb Fetlock Joint Motion (Kinematics) | Trot, in-hand trot | Holmstrom et al. 1994, Morales et al. 1998 | Trot: increased flexion during stance. In-hand trot: greater extension at lift-off. |
| Scapula Segment Motion (Kinematics) | Trot, in-hand trot, trot (treadmill) | Back et al. (1994), Holmstrom et al. (1994), Morales et al. (1998) | Greater ROM and segment inclination |
| Femur Segment Motion (Kinematics) | Trot | Holmstrom et al. 1994 | Increased inclination during stance |
| Pelvis Segment Motion (Kinematics) | Trot | Holmstrom et al. 1994 | Decreased inclination during stance |
| Hindlimb Pro/ Retraction Angular Velocity (Kinematics) | Trot | Holmstrom et al. 1997 | Not described |
| Hindlimb Fetlock Joint Angular Velocity (Kinematics) | Trot | Holmstrom et al. 1997 | Not described |
| Tarsal Joint Angular Velocity (Kinematics) | Trot | Holmstrom et al. 1997 | Faster during stance |
| Pelvis Segment Angular Velocity (Kinematics) | Trot | Holmstrom et al. 1997 | Nearly zero in poor horses and faster in good horses. |
| Forelimb Hoof Trajectory (Kinematics) | Trot | Holmstrom et al. 1994 | Greater FL hoof trajectory |
| **TRUNK MOTION** | | | |
| Dorsoventral displacement (Accelerometer) | Walk, trot, canter | Biau and Barrey, 2004 | Increased |
| Dorsoventral activity (Accelerometer) | Walk, trot, canter-trot transition | Biau et al. (2002), Biau and Barrey (2004) | Increased |
| Averaged energy variation (Accelerometer) | Trot-halt transition, canter-halt transition | Biau et al. (2002) | Decreased |
| Averaged frequency (Accelerometer) | Trot-walk transition, canter-halt transition | Biau et al. (2002) | Decreased |
| Averaged frequency variation (Accelerometer) | Canter-trot transition | Biau et al. (2002) | Decreased |
| Frequency fluidity (Accelerometer) | Canter-trot transition, canter-walk transition, canter-halt transition | Biau et al. (2002) | Increased |
| Energy fluidity (Accelerometer) | Walk-halt transition | Biau et al. (2002) | Increased |
| **IMPULSION** | | | |
| Vector of propulsion (Accelerometer) | Walk, trot, canter | Biau and Barrey, 2004 | Increased |
| Vector of breaking (Accelerometer) | Walk, trot | Biau and Barrey, 2004 | Increased |
| Longitudinal activity (Accelerometer) | Trot, canter | Biau and Barrey, 2004 | Increased |
| Breaking deceleration (Accelerometer) | Trot-halt transition, canter-walk transition | Biau et al. (2002) | Increased deceleration |
| **STRIDE LENGTH/ADJUSTABILITY** | | | |
| Stride length (Kinematics) | Extended canter (left lead), extended trot | Deuel and Park (1990a, b) | Longer stride length |
| Stride Velocity (Kinematics) | Extended canter (left lead), extended trot | Deuel and Park (1990a, b) | Faster velocity |
| FL Step Length (TrF -> LdF) (Kinematics) | Extended canter (left lead), two-stride lead change (pre-lead change stride) | Deuel and Park (1990a) | Decreased FL step length |
| HL Step Length (TrH -> LdH) (Kinematics) | Extended canter (left lead) | Deuel and Park (1990a) | Longer HL step length |
| Mid Step Length (LdH -> TrF) (Kinematics) | Extended canter (left lead), two-stride lead change (pre-lead change stride) | Deuel and Park (1990a) | Longer mid-step length |
| Airborne Step (LdF -> TrH) (Kinematics) | Two-stride lead change (pre and post-lead change stride) | Deuel and Park (1990a) | Longer airborne step |
| **CONNECTION** | | | |
| Mean Rein Tension (Rein tension meter) | Entire dressage training test | von Borstel et al. (2014) | Decreased mean rein tension |
| Max Rein Tension (Rein tension meter) | Entire dressage training test | von Borstel et al. (2014) | Decreased max rein tension |
| Variance of Rein Tension (Rein tension meter) | Entire dressage training test | von Borstel et al. (2014) | Decreased rein tension variability |
| Key: FL Forelimb, HL Hindlimb, TrF Trailing forelimb, LdF Leading forelimb, TrH Trailing hindlimb, LdH Leading hindlimb, ROM Range of motion. | | | |
