# Supplementary material for: A scoping review of determinants of performance in dressage
Source: PeerJ. 2020 Apr 24;8:e9022. doi: 10.7717/peerj.9022 (PMC7185025; doi:10.7717/peerj.9022)
Supplement: Table S9 [file peerj-08-9022-s009.docx]

Supplemental Table S8: Significant rider performance outcome measures

| **OUTCOME MEASURES**  **(Measurement tool)** | **Gait/**  **Movement** | **Articles** | **Advanced/ Elite Rider** | **Novice/ Non-rider** | **Significant Performance Effect** |
| --- | --- | --- | --- | --- | --- |
| **RANGE OF MOVEMENT** | | | | | |
| TRUNK SEGMENT FLEXION/ EXTENSION, A/P, PITCH (Kinematics, IMU) | Sitting Trot | Alexander et al. (2015)  Bystrom et al. (2009)  Eckardt et al. (2014)  Eckardt and Witte (2016)  Terada et al. (2006) | 10.12 ± 4.65 | 9.5 ± 2.3 | Beginner riders max and mean values showed significantly (max p=0.026, mean p=0.04) more forward trunk angle than pro riders (Eckardt and Witte, 2016). |
| TRUNK SEGMENT LATERAL FLEXION, M/L, ROLL (Kinematics, IMU) | Canter | Eckardt and Witte (2016) | 4.4 ± 1.8 | 7.0 ± 3.2 | Beginner riders had significantly (p<0.05) higher ROM than pro riders (i.e. less stable trunk) (Eckardt and Witte, 2016). |
| HEAD SEGMENTAL ANGLE, A/P, PITCH (Kinematics, IMU) | Sitting Trot | Bystrom et al. (2009) Eckardt et al. (2014)  Eckardt and Witte (2016) | 12.9 ± 2.42 | 11.6 ± 5.2 | Minimum values showed that beginner riders tilt their head significantly (p=0.04) more forward than pro riders (Eckardt and Witte, 2016). |
| PELVIC ANTERIOR/ POSTERIOR TILT, PITCH (Kinematics, IMU) | Walk | Bystrom et al. (2010)  Munz et al. (2014) | 10.40 ± 0.99 | 8.1 ± 4.1 | Pro riders had significantly (p<0.05) greater min values (pelvis more neutral ROM) (Munz et al, 2014) |
|  | Sitting Trot | Alexander et al. (2015)  Bystrom et al. (2009)  Munz et al. (2014)  Munz et al. (2013)  Eckardt et al. (2014)  Eckardt and Witte (2016) | 12.40 ± 1.97 | 12.90 0.85 | Beginner riders had significantly (p<0.05) lower values for min and max (i.e. pro pelvis more neutral ROM) (Munz et al, 2014). |
|  | Canter | Munz et al. (2014) Eckardt and Witte (2016) | 21.0 ± 4.24 | 22.65 ± 0.64 | Pro showed significantly (p<0.05) lower max values than beginner riders (pro pelvis more neutral ROM) (Munz et al., 2014). |
| PELVIC LATERAL FLEXION, M/ L, ROLL (Kinematics, IMU) | Sitting Trot | Alexander et al. (2015) Bystrom et al. (2009)  Munz et al. (2013)  Munz et al. (2014)  Eckardt et al. (2014)  Eckardt and Witte (2016) | 4.4 ± 1.22 | 5.85 ± 0.92 | ROM significantly (p<0.05) greater for beginner riders than pro riders (Munz et al., 2014). |
| ELBOW JOINT ANGLE (Kinematics, IMU) | Sitting Trot | Terada et al. (2006) Eckardt et al. (2014)  Eckardt and Witte (2016) | 16.34 ± 4.73 | 11.80 ± 0.99 | Significantly (p=0.02) greater ROM for left elbow in pro riders with pro riders showing significantly (p<0.01) greater elbow flexion (Eckardt and Witte, 2016). |
| KNEE JOINT ANGLE (IMU) | Sitting Trot | Eckardt et al. (2014)  Eckardt and Witte (2016) | 6.00 ± 0.81 | 10.35 ± 0.78 | Pro riders show significantly (p<0.01) less knee joint ROM than beginner riders (Eckardt and Witte, 2016). |
|  | Canter | Eckardt and Witte (2016) | 9.80 ± 1.98 | 11.85 ± 0.49 | Pro riders show significantly (p<0.05) less left knee ROM than Beginner riders (Eckardt and Witte, 2016). |
| **PHYSIOLOGICAL DEMANDS OF RIDING** | | | | | |
| MEAN HEART RATE (BPM) (Gas Analyser, Heart Rate Monitor) | Walk | de Barro Souza et al. (2008) Devienne et al. (2000)  Westerling (1983)  Meyers (2006)  Sung et al. (2015) | 101.8 ± 9.14 | 105.0 ± 23.57 | Elite riders had significantly (p=0.021) lower HR during walk than amateurs (Sung et al., 2015). |
|  | Recovery 5 min Following Riding | Sung et al. (2015) | 102.8 ± 12.67 | 123.44 ± 5.71 | Elite riders had significantly (p<0.001) lower HR during recovery than amateurs (Sung et al., 2015). |
| **COORDINATION (HORSE AND RIDER)** | | | | | |
| ELBOW - HORSE DISCRETE RELATIVE PHASE (°) (Kinematics) | Mechanical Horse at Varying Oscillation Frequencies | Baillet et al. (2017) | 6.5 ± 21.2 | 32.5 ± 72.4 | Significant (p<0.05) effect of expertise with riders maintaining in-phase movement across all frequencies compared to non-riders. Non-riders were only in-phase during the slowest frequency (Baillet et al., 2017). |
| TRUNK - HORSE DISCRETE RELATIVE PHASE (°)(Kinematics) | Mechanical Horse at Varying Oscillation Frequencies | Baillet et al. (2017) | 178 ± 22.9 | 174.9 ± 29.2 | Significant (p<0.05) effect of expertise with riders oscillating out-of phase with horse across all frequencies compared to non-riders (Baillet et al., 2017). |
| HEAD - HORSE DISCRETE RELATIVE PHASE (°)(Kinematics) | Mechanical Horse at Varying Oscillation Frequencies | Baillet et al. (2017) | 46.9 ± 58.7 | 95.6 ± 62.2 | Riders head oscillations were significantly different (p<0.05) from non-riders and moved out of phase for V0 and in-phase for V1 - V3, but non-riders were only in- phase in V3 (Baillet et al., 2017). |
| HEAD - HORSE DISCRETE RELATIVE PHASE VARIABILITY (°)(Kinematics) | Mechanical Horse at Varying Oscillation Frequencies | Baillet et al. (2017) | 58.7 | 62.2 | Head segment was significantly (p<0.05) less variable in non-riders at low oscillation frequency (V0) compared to riders (Baillet et al., 2017). |
| Average Deviation of Length of Vector (Kinematics) | Sitting Trot | Peham et al. (2001) | 11.5 ± 1.4% | 13 ± 2.8% | Motion of the pro rider and horse were found to be more consistent, as shown by similarities in ridden and unridden trot speeds, significantly (p<0.05) higher dressage scores and significantly (p<0.05) lower average deviation of LV (Peham et al., 2001). |
| **COORDINATION (RIDER)** | | | | | |
| MEAN RELATIVE PHASE (LUMBAR - CERVICAL) (A/P) (degrees) (IMU) | Simulated gallop (included in canter) | Oliver et al. (2017) | 153.99 ± 22.67 | 188.27 ± 24.24 | Significant difference (p=0.009) between professional and club riders (Oliver et al., 2017). |
| Key: A/P Anterior/Posterior, IMU Inertial measurement unit, ROM Rage of motion, Pro Professional, BPM Beats per minute, HR eart rate, | | | | | |
