# Supplementary material for: A scoping review of determinants of performance in dressage
Source: PeerJ. 2020 Apr 24;8:e9022. doi: 10.7717/peerj.9022 (PMC7185025; doi:10.7717/peerj.9022)

**Theoretical model reasoning (Figure 2)**

The following texts are extracts from the manuscript to show the reasoning behind links between horse theme and rider objective measures that are significantly influenced by skill. Significant rider measures are listed in accordance with Figure 3 and separated by gait. Text is coloured by horse theme.

**Walk**

**Lower heart rate**

When comparing the physiological demands for the athlete, a significant increase in heart rate in amateur athletes during two minutes of walking compared to skilled athletes was attributed to differences in physical fitness by Sung et al. (2015) as their resting heart rates were also significantly different. It is interesting to note that the heart rate increase at walk compared to resting values was 28.9% in amateur athletes and 14.1% in skilled athletes, but when practicing jumping the increases over resting values were 73.5% and 84.7%, respectively. This could indicate that skilled athletes do not work as hard during walking, due to developing better relaxation and/or harmony with the horse. Münz et al. (2014) suggested that the pelvis of less skilled athletes moves ‘‘ahead’’ of the horses’ movement and Wolframm et al. (2013) found lower interclass correlations between horse and rider motion in walk compared to canter. Out of phase timing of the rider with the horse may disrupt the rhythm of the horse and result in greater energy expenditure for the less skilled athlete to maintain an active walk (active walk).


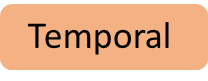

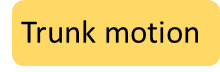

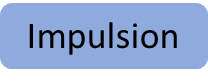


**Greater forward pelvic tilt**

Since the walk does not have suspension phases, back movements and pitching rotations of the horse’s trunk are driven by the limb movements (Faber et al., 2000; Byström et al., 2010).

Pelvic rotations occur twice per stride and, movements of the rider and the horse are less well synchronized than for the other gaits (Wolframm et al., 2013). Comparing skilled riders with beginners, Münz et al. (2014) found that the pelvis underwent greater anterior to posterior pelvic motion (referred to in the paper as forward pelvic tilt) in skilled riders compared with beginners and this was associated with increased nose-up trunk rotation of the horse.


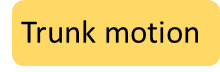

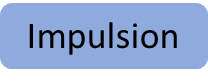


**Trot**

**Lower average deviation of Length of Vector**

Muscular strength determines the development of propulsive force; more powerful muscles are able to generate force more rapidly which is evident in a shorter stance duration (Back et al., 1994). Shorter stance durations were identified as having a positive influence on stride quality both in the forelimbs (Deuel and Park, 1990a; Deuel and Park, 1990b) and hindlimbs (Holmström et al., 1994) resulting in smaller duty factors (Holmström et al., 1994). Swing durations are influenced by both stride duration and stance duration. Deuel and Park (1990b) found that shorter hind limb and longer forelimb swing durations were objective measures of performance. Taken together, having relatively shorter stance phases and relatively longer swing phases is aesthetically pleasing because the trot appears less grounded and more bouncy, which is regarded positively by judges would alsoand explains the greater dorsoventral displacement and activity found by Biau and Barrey (2004).

Skilled riders are said to have a stabilizing effect on the horse, as shown by a reduction in motion pattern variability (Peham et al., 2004). Improved rider-horse harmony will reduce disruption in temporal variables and dorsoventral motion associated with horse performance (Wolframm et al., 2013).


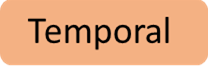

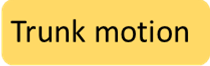

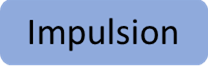


**Less pelvic lateral flexion**

In skilled riders, the pelvis rotates from anterior to posterior tilt over the stride cycle with a smaller amount of lateral tilt (Münz et al., 2014)…..

The posture of the pelvis and upper body segments dictates how pressure is distributed under the saddle (de Cocq et al., 2009; Gunst et al., 2019), which affects the aids communicated to the horse and also impacts on the horses’ balance (de Cocq et al., 2010b). In skilled riders, pelvic motion is independent of trunk, head or other segment motion, which requires dynamic postural control (Engell et al., 2016). When the rider achieves an advanced level of dynamic postural control, it improves the harmony between horse and rider (Peham et al., 2001; Münz et al., 2014), and translates to higher average dressage scores (Peham et al., 2001).


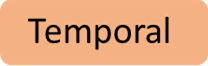

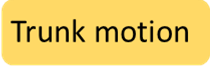

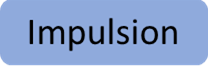


**Reduced forward trunk pitch**

….whilst the trunk maintains a more consistent vertical posture…

The challenge for the rider is therefore often associated with their ability to maintain dynamic postural control and harmony with the horse whilst coping with the large vertical and longitudinal accelerations and decelerations of the horse’s trunk in trot (Terada, 2000; Byström et al., 2015).

The posture of the pelvis and upper body segments dictates how pressure is distributed under the saddle (de Cocq et al., 2009; Gunst et al., 2019), which affects the aids communicated to the horse and also impacts on the horses’ balance (de Cocq et al., 2010b).

In skilled riders, pelvic motion is independent of trunk, head or other segment motion, which requires dynamic postural control (Engell et al., 2016). When the rider achieves an advanced level of dynamic postural control, it improves the harmony between horse and rider (Peham et al., 2001; Münz et al., 2014), and translates to higher average dressage scores (Peham et al., 2001). Skilled riders control body position by coordinating activity level and antagonistic timing of Erector Spinae and Rectus Abdominis muscles (Terada, 2000; Pantall et al., 2009), whilst novice riders display energetically inefficient co-activation of Erector Spinae and Rectus Abdominis muscles (Pantall et al., 2009) and use Adductor Magnus to stabilize the trunk (Terada, 2000). Phasic activity in Rectus Abdominis in mid-stance is used to stabilize the rider’s trunk and enable the rider to follow the horse’s movement by rotating the pelvis posteriorly as the horse’s body reverses direction from downward to upward motion (Terada et al., 2004; Pantall et al., 2009).


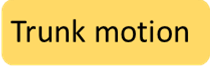

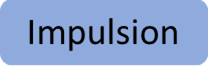


**Greater forward pelvic tilt**

Again, pelvic motion from anterior to posterior tilt of the rider was found to significantly increase nose up trunk tilt of the horse during trotting (Münz et al., 2014).

The challenge for the rider is therefore often associated with their ability to maintain dynamic postural control and harmony with the horse whilst coping with the large vertical and longitudinal accelerations and decelerations of the horse’s trunk in trot (Terada, 2000; Byström et al., 2015).

The posture of the pelvis and upper body segments dictates how pressure is distributed under the saddle (de Cocq et al., 2009; Gunst et al., 2019), which affects the aids communicated to the horse and also impacts on the horses’ balance (de Cocq et al., 2010b).


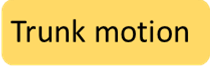

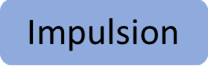


**Less forward head tilt**

….the head a more consistent and stiller horizontal posture (Eckardt and Witte, 2016).

The challenge for the rider is therefore often associated with their ability to maintain dynamic postural control and harmony with the horse whilst coping with the large vertical and longitudinal accelerations and decelerations of the horse’s trunk in trot (Terada, 2000; Byström et al., 2015).


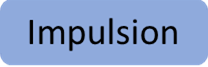


**Less knee flexion-extension ROM**

Less knee flexion-extension ROM in skilled riders may also relate to the rider’s ability to cope with the motion of the horse, with less of a tendency in skilled riders to pull up the knees in an effort to remain balanced (Byström et al., 2015).

A stiller leg will improve the rider’s ability to provide consistent and precise aids to the horse, resulting in more finite speed, gait and/or movement changes.


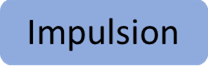

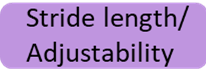


**More left elbow flexion and elbow flexion-extension ROM**

The ability to maintain consistent contact with the bit at all gaits is also necessary to facilitate good rider-horse communication (Eisersiö et al., 2013; von Borstel and Glißman, 2014). Perturbations due to the motion of the horse’s trunk are accommodated by the rider with the apparent goal of allowing the rider’s hand to maintain a consistent position relative to the bit (Terada et al., 2006; Eisersiö et al., 2013). Eckardt and Witte (2016) reported an increase in flexion-extension ROM of the elbow in skilled riders to effect this.

**Note: Joint/Segment kinematics are specifically related to head and neck posture of the horse for this exercise, as joint and segment kinematics changes will influence objective measures in all themes.**


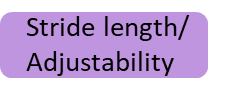

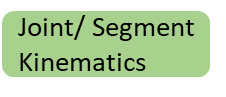


**Canter**

**Lower mean relative phase (lumbar/cervical)**

As such, heightened pelvic mobility and postural control are required by the rider to maintain balance and harmony with the horse (Olivier et al., 2017), although greater synchronicity is possible due to canter being a three-beat gait (Wolframm et al., 2013).

In skilled riders, pelvic anterior-posterior ROM (Münz et al., 2014) trunk lateral bending ROM and left knee flexion-extension ROM (Eckardt and Witte, 2016) are smaller compared to less skilled riders. As trunk ROM of the horse in pitch and longitudinal forces increase, a closer coupling of the pelvis in anterior-posterior tilt and greater control of the upper body are required in order to follow the phasic motions of the horse (Lovett et al., 2005; Wolframm et al., 2013; Münz et al., 2014).


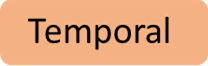

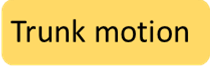

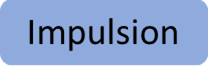


**Less trunk lateral bending ROM**

In skilled riders, pelvic anterior-posterior ROM (Münz et al., 2014) trunk lateral bending ROM and left knee flexion-extension ROM (Eckardt and Witte, 2016) are smaller compared to less skilled riders. As trunk ROM of the horse in pitch and longitudinal forces increase, a closer coupling of the pelvis in anterior-posterior tilt and greater control of the upper body are required in order to follow the phasic motions of the horse (Lovett et al., 2005; Wolframm et al., 2013; Münz et al., 2014).

A reduction in rider trunk lateral bending ROM is likely to reduce amplification of the asymmetry of the gait and improve medio-lateral and rotational stability in the horse (Symes and Ellis, 2009).


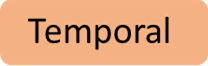

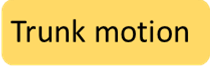

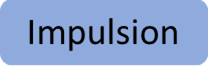


**Less pelvic flexion-extension ROM**

In skilled riders, pelvic anterior-posterior ROM (Münz et al., 2014) trunk lateral bending ROM and left knee flexion-extension ROM (Eckardt and Witte, 2016) are smaller compared to less skilled riders. As trunk ROM of the horse in pitch and longitudinal forces increase, a closer coupling of the pelvis in anterior-posterior tilt and greater control of the upper body are required in order to follow the phasic motions of the horse (Lovett et al., 2005; Wolframm et al., 2013; Münz et al., 2014).


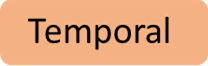

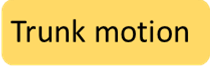

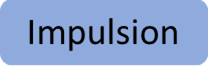


**Less left knee flexion-extension ROM**

As with trot, a stiller left leg is likely to improve communication with the horse, probably reflects better rider balance (Byström et al., 2015; Eckardt and Witte, 2016) and is expected to disrupt canter with a right lead more than a left lead (Symes and Ellis, 2009).


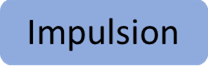

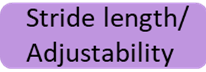

Supplement: Text S1 — The following texts are extracts from the article to show the reasoning behind links between horse theme and rider objective measures that are significantly influenced by skill. Significant rider measures are listed in accordance with Figure 2 and separated by gait. Text is coloured by horse theme. [file peerj-08-9022-s011.docx]
